# Evaluation of Circulating Chitotriosidase Activity in Children with Obesity

**DOI:** 10.3390/jcm11133634

**Published:** 2022-06-23

**Authors:** Ioana Țaranu, Mihaela Iancu, Cecilia Lazea, Camelia Alkhzouz, Nicoleta Răcătăianu, Cristina-Sorina Cătană, Andreea-Manuela Mirea, Diana Miclea, Sorana D. Bolboacă, Cristina Drugan

**Affiliations:** 1 Department of Medical Informatics and Biostatistics, Iuliu Hațieganu University of Medicine and Pharmacy, Louis Pasteur Str., No. 6, 400349 Cluj-Napoca, Romania; taranu.ioana@umfcluj.ro (I.Ț.); miancu@umfcluj.ro (M.I.); sbolboaca@umfcluj.ro (S.D.B.); 2 Pediatric Clinic 1, Emergency Pediatric Hospital, Calea Moților, No. 68, 400370 Cluj-Napoca, Romania; mirea.andreea.manuela@elearn.umfcluj.ro; 3 Department Mother and Child, Iuliu Hațieganu University of Medicine and Pharmacy, Calea Moților, No. 68, 400370 Cluj-Napoca, Romania; calkhuzouz@umfcluj.ro; 4 Integrated Ambulatory of Endocrinology, Infectious Diseases Clinical Hospital, Calea Moților, No. 19, 400000 Cluj-Napoca, Romania; comanniko@yahoo.com; 5 Department of Medical Biochemistry, Iuliu Haţieganu University of Medicine and Pharmacy Cluj-Napoca, Louis Pasteur Str., No. 6, 400349 Cluj-Napoca, Romania; ccatana@umfcluj.ro (C.-S.C.); cdrugan@umfcluj.ro (C.D.); 6 Department of Molecular Sciences, Iuliu Hațieganu University of Medicine and Pharmacy, Louis Pasteur Str., No. 6, 400349 Cluj-Napoca, Romania; diana.miclea@umfcluj.ro

**Keywords:** inflammation, Body Mass Index (BMI), human chitotriosidase (CHIT1), 24 bp duplication (*dup24*), *G102S* polymorphism, macrophage activation, obesity-driven inflammation

## Abstract

Childhood obesity progresses to metabolic disturbances via low-grade inflammation. Identifying novel molecules that reflect the activity of the immune responses is critical in understanding its underlying pathogenesis. Our exploratory study aimed to evaluate the change of chitotriosidase (CHIT1) plasma activity according to Body Mass Index (BMI)-for-age z score in pediatric patients. The study evaluated 68 children consisting of 47.1% girls with a mean age of 12.47 ± 3.71 years and 52.9% boys with a mean age of 11.93 ± 3.18 years. The effect of the most frequent CHIT1 gene variants, the 24 base pair duplication (*dup24*) and *G102S* polymorphism, upon the association between circulating CHIT1 activity and the obesity level, was also investigated. A significantly higher logCHIT1 plasma activity was found in children with extreme obesity than in children with overweight (*p* = 0.048 for the uncorrected CHIT1 and 0.026 for the corrected CHIT1). The BMI-for-age z score significantly (*p* = 0.031) predicts increased CHIT1 activity in children with overweight, obesity, and extreme obesity after controlling for the two gene variants, age, gender, and time since weight gain. *Dup24* and *G102S* polymorphism were significant independent predictors (*p*-values < 0.002) for the change of CHIT1 plasma activity. Circulating CHIT1 might be an accurate indicator of inflammation in children with obesity. Its role and the effect of the *dup24* and *G102S* variants on the CHIT1 activity should be validated in a larger cohort.

## 1. Introduction

Chronic low-grade inflammation is a process that links obesity with metabolic disturbances via macrophage polarization and activation of the acquired immune cells [1,2]. A high level of obesity results in a 17-fold increase in the cardiovascular risk in children and leads to obesity-driven complications such as metabolic syndrome and type 2 diabetes mellitus [3,4]. In clinical practice, it is a challenge to find indicators and biomarkers for the severity of childhood obesity [5]. Since obesity in children predicts the onset of cardiovascular disease and type 2 diabetes mellitus in adulthood [6,7], the study of such indicators is clinically relevant. Body Mass Index (BMI)-for-age z score, waist, hip circumference, and waist-to-height ratio are anthropometric measures currently used in clinical and research settings for the diagnosis of obesity, screening of the metabolic syndrome, and evaluation of strategies for weight management in the pediatric population [8,9,10,11]. Classical inflammatory markers used in daily practice include mainly paracrine and endocrine regulators secreted by the adipose tissue and are associated with metabolic complications in children with obesity [12,13].

Human chitotriosidase (CHIT1) is an enzyme synthesized by neutrophils, adipose tissue-residing monocytes and activated macrophages [14,15]. The circulating CHIT1 activity is a valuable marker in lysosomal storage disorders (i.e., Gaucher disease type 1) [16,17]. The immunomodulatory effects of CHIT1 implicate polarization of macrophages and activation of immune cells involved in the acquired immunity [15,18]. Adipose tissue inflammation in obesity is initially mediated by local proliferation of the residing pro-inflammatory macrophages [19], followed by the secretion of chemoattractants (mainly monocyte chemoattractant protein-1 (MCP-1)) by the activated macrophages. The MCP-1 further increases the recruitment of more monocytes and macrophages into the adipose tissue [20,21]. The level of CHIT1 mRNA proved to be associated with MCP-1 mRNA level in adipose tissue. In adults, it is more elevated in patients with overweight with and without type 2 diabetes mellitus [14]. High CHIT1 activity is also reported in patients with conditions associated with obesity, such as atherosclerosis and non-alcoholic fatty liver disease [22,23].

In a population-based study conducted in a Brazil, Tamanaha et al. [24] reported higher CHIT1 serum levels in adults with obesity than in adults with normal BMI. Increased plasma CHIT1 levels were also reported in children with immunodeficiency and lysosomal storage disorders [25,26]. Kabaroğlu et al. reported higher plasma CHIT1 levels in children with obesity and impaired glucose tolerance than those without impaired glucose tolerance [23]. This evidence might legitimate the use of CHIT1 circulating activity as a biomarker for subclinical inflammation in childhood obesity.

Grace et al. [27] and Csongrádi et al. [28] recommended evaluating the genetic variants affecting CHIT1 gene expression when CHIT1 activity is measured. The CHIT1 gene is located on the 1q31-1q32 chromosome and consists of 12 exons, spanning about 20 kb [29,30]. Two common allelic variants, *dup24* and *G102S*, are frequently reported in the European population as potential sources of plasma CHIT1 levels variation [31]. *dup24* is a 24 base pair duplication in exon 10 (CHIT1(NM_003465.3):c.1049_1072dup (p.Trp358Ter); rs3831317) and *G102S* is a 304 G-A transition in exon 4 resulting in a Gly102-to-Ser substitution (CHIT1(NM_003465.3):c.304G > A (p.Gly102Ser); rs2297950). The common *dup24* variant, which is recessively inherited, leads to alternate splicing and in-frame deletion of 87 nucleotides via activation of a cryptic 3′ splice site in exon 10 causing an absence of a functional enzyme in patients homozygous for the mutant allele [30]. The prevalence of CHIT1 deficiency varies according to geographical region and is estimated at 6% to 10% in the European population [30,32].

The 4-methylumbelliferyl-(4-deoxy)chitobiose, a substrate for the measurement of enzymatic activity in adults, due to its ability to prevent transglycosylation, shows higher accuracy in the quantification of chitotriosidase levels than 4-methylumbelliferyl-chitotrioside (4-MU-3C) [33,34,35]. *G102S*, a guanine to adenine transition at position 304 of the cDNA, can lead via potential transglycosylation of the CHIT1 catalytic domain to a reduced catalytic efficiency towards the 4-methylumbelliferyl-chitotrioside substrate and subsequently to a reduced CHIT1 activity [27,33]. In vitro functional studies showed that the expression of the *G102S* allele results in a 4- to 5-fold reduction in CHIT1 catalytic efficiency compared to the activity of the wild type enzyme [27]. To address this limitation, Bussink et al. [34] proposed a correction of plasma CHIT1 activity in adult patients heterozygous and homozygous for the *G102S* allele when using 4-methylumbelliferyl-chitotrioside as substrate. More specifically, a multiplying factor of 1.3 and 1.6 in heterozygotes and homozygotes for the *G102S* allele, respectively, except for the cases where patients carry a concomitant *dup24* allele, reflects more accurately the CHIT1 enzymatic activity [34]. To the best of our knowledge, the influence of these two CHIT1 gene variants on the plasma CHIT1 activity in children with obesity has not been investigated and reported.

We hypothesize that plasma CHIT1 activity, as a marker of low-grade systemic inflammation, might be elevated in children with high levels of obesity. The objectives of the current study were to assess in children: (i) The variability of CHIT1 plasma activity by weight status based on BMI-for-age z scores; (ii) The association of *dup24* and *G102S* polymorphism with plasma CHIT1 activity in patients with different levels of obesity by analyzing the observed difference in enzymatic activity between genotype subgroups and the effect of the Bussink et al. [34] correction method; (iii) The contribution of *dup24*, *G102S* polymorphism and BMI-for-age z score to the variation of CHIT1 plasma activity.

## 2. Materials and Methods

The study has been approved before patient enrollment by the Iuliu Hațieganu University of Medicine and Pharmacy Ethics Committee (approval no. 179/30.05.2019) and the Ethics Committee of the Children′s Emergency Hospital Cluj-Napoca (approval no. 71/20.06.2019).

Children provided assent for participation and their parents or legal representatives signed the written informed consent before their enrollment. The study information was adapted according to the age categories of 5 to 11 and 12 to 18 years old. 

### 2.1. Patients and Clinical Variables

A cross-sectional exploratory study with consecutive data collection was conducted between September 2019 and July 2020 and included children receiving inpatient and outpatient care in the Pediatric Clinic 1, Medical Genetics Unit from the Children’s Emergency Hospital and the Endocrinology Department of the Infectious Disease Hospital in Cluj-Napoca, Romania.

Children aged 5 to 18 years diagnosed with overweight or obesity (diagnosis codes E66.0 or E66.9 according to the 10th revision of the International Statistical Classification of Diseases and Related Health Problems) as cases and normal-weight children as controls were eligible for the study. Children with syndromic obesity, those with acute or chronic know inflammatory diseases (i.e., juvenile arthritis, thyroiditis with hypo- or hyperthyroidism), metabolic syndrome, or infectious diseases (i.e., acute upper or lower respiratory tract diseases, urinary tract infections or gastroenteritis, human C-reactive protein serum concentration (hCRP) ≥ 10 mg/dL and positive pharyngeal exudate, urine test or stool cultures, etc.) were excluded. Syndromic obesity was defined as the presence of dysmorphic features, organ malformations, hyperphagia, or cognitive delay in obese children with or without detected mutations or chromosomal abnormalities where, in most cases, an appropriate assent was not possible [36]. Previous studies had indicated changes in the CHIT1 activity in the presence of acute infections [37,38,39,40] and chronic inflammatory diseases [41,42,43,44,45]. Children homozygotes for *dup24* who presented no CHIT1 activity [27,30] were also excluded.

The control group consisted of normal-weight children who came consecutively for a routine examination or a suspected but ruled out (after medical evaluation and investigations) endocrine disturbance. For each child with overweight or obesity included in the study, we invited a child with normal weight within the same gender and age group (5 to 11 years old and 12 to 18 years old). Only children who assented to participate and whose parents agreed by signing the informed consent were included in our study.

The anthropometric assessment included weight and height using a stadiometer and a mechanical beam scale to ± 0.1 cm and ± 0.1 kg, respectively, after an 8-h fasting period. The z scores for grouping the patients as normal weight (<1 BMI-for-age z score), overweight (≥1 BMI-for-age z score), obese (≥2 BMI-for-age z score), and extremely obese (≥3 BMI-for-age z score) were calculated using the World Health Organization (WHO) Anthroplus application v1.0.4. We used the definition of the z-score according to the 2006 WHO Child Growth Standards report as “*the deviation of an individual’s value from the median value of a reference population, divided by the standard deviation of the reference population (or transformed to normal distribution)*” [46]. During the medical examination, parents reported the time since weight gain in their children.

The pubertal staging was evaluated by a pediatric endocrinologist based on the development of pubic hair and breast volume in girls, and the testicular volume in boys, according to the Marshall and Tanner stages [47,48]. Children included in the study were classified as prepubertal (Tanner stage 1), pubertal (Tanner stages 2–4), or postpubertal (Tanner stage 5).

The following laboratory measurements were also collected for each child included in the study: total absolute white blood cells count (WBC), human C-reactive protein (hCRP), ferritin, and erythrocyte sedimentation rate (ESR). 

### 2.2. Plasma CHIT1 Assay

Samples of three mL blood were collected in EDTA vacutainers, followed by centrifugation (10 min at 4000 rpm) at 4 °C. Plasma was separated in the upper phase and stored at −20 °C until further analysis. Plasma CHIT1 activity was measured by a fluorometric method, using the artificial substrate 4-methylumbelliferyl-chitotrioside, according to the method described by Hollak et al. [49]. CHIT1 plasma activity was expressed as nanomoles of substrate hydrolyzed per milliliter per hour (nmol/mL/h). Based on adult and healthy pediatric subjects, reference values in our laboratory ranged between 3 and 100 nmol/mL/h.

### 2.3. CHIT1 Genotyping

Genomic deoxyribonucleic acid (DNA) was isolated from 2 mL whole blood using the Wizard Genomic purification kit according to the manufacturer’s protocol (Promega Corporation, Madison, WI, USA) [50]. A working concentration above 50 ng/µL (Nanodrop 1000 Spectrophotometer, Thermo Fisher Scientific, Waltham, MA, USA) was set as a criterion for the genetic analysis. Specific primers (Thermo Scientific, Santa Clara, CA, USA) [51] were used to amplify fragments of 195 and 219 base pairs in exon 10 (Table 1). Polymerase chain reaction (PCR) was followed by electrophoretic separation of the amplified DNA on MetaPhor^TM^ 1.5% agarose gel (for fine separation and resolution of small nucleic acids) and visualization by RedSafe™ Nucleic Acid Staining Solution (20,000×) (iNtRON Biotechnology, Burlington, MA, USA) with a 50 basepair DNA ladder (Thermo Scientific, Santa Clara, CA, USA).

The genotypes were confirmed by Sanger sequencing. The PCR reaction was repeated using the same primers. The presence of the PCR products was verified by gel electrophoresis and then the PCR products were purified with ExoSap-IT ^TM^ (Applied Biosystems, Thermo Fisher Scientific, Waltham, MA, USA). The bidirectional sequencing was performed using BigDye^TM^ Terminator v3.1 (Applied Biosystems, Thermo Fisher Scientific, Waltham, MA, USA). The obtained sequencing products were purified with Dynabeads^®^ Sequencing Clean-Up kit (Invitrogen, Thermo Fisher Scientific, Waltham, MA, USA). Finally, the capillary electrophoresis on Applied Biosystems 3500 Analyzer (Applied Biosystems, Thermo Fisher Scientific, Waltham, MA, USA) was performed and the results were visualized with Unipro Ugene software [52]. Boot et al. [30] reported a third band on the electrophoresis representing a hybrid DNA molecule formed between the 195 bp and 219 bp DNA fragments during the PCR reaction.

Amplification and detection of the *G102S* polymorphism was performed by Real-time PCR Allelic Discrimination on Real-time PCR Thermal Cycler (Applied Bio systems), using a pre-designed TaqMan single nucleotide polymorphism (SNP) assay mix (Applied Biosystems (Assay ID: C_160346_1_)). The final Real-time PCR mixture was a 10 µL volume containing 5.875 µL PCR grade water, 2.5 µL Taqman genotyping Master Mix, 0.125 µL Taqman solution and 0.5 µL 0.11% bovine serum albumin solution. One no template control sample was used for each analysis set. The followed protocol included the steps: 30 min at 60 °C and 10 min at 95 °C followed by a cycling phase consisting of 40 cycles (step 1 of 15 min at 95 °C and a step 2 at 60 °C for 1 h), and a post-PCR read of 30 min at 60 °C. The wild C and mutant T alleles were identified based on allelic discrimination and amplification plots (StepOne Software version 2.3, Applied Biosystems, Thermo Fisher Scientific, Waltham, MA, USA).

Correction of measured plasma CHIT1 activity was made according to Bussink et al. [34]. No correction was applied for *dup24* heterozygotes because the used method could not allow determining whether the two variants were or were not on the same allele.

### 2.4. Statistical Analysis

Statistical analysis was performed R software v.4.1.1. [53]. Categorical variables were expressed as absolute and relative frequency (n, %). The fit of empirical distribution with univariate normal distribution was performed by descriptive statistics, Shapiro–Wilk’s test, or estimation of distribution parameters by maximizing the likelihood function. Quantitative variables that followed the Gaussian distribution were summarized as arithmetic mean and standard deviation, while those with deviations from normal distribution were reported as the median and interquartile range (IQR, Percentile 25—Percentile 75) and minimum to maximum values. The CHIT1 values were expressed on the logarithmic scale (log10), and the transformed values proved normally distributed. The geometric mean (accompanied by 95% confidence interval, CI)) and standard deviation were used to report the empirical distribution of log-transformed CHIT1 plasma activity.

Chi-Squared or Fisher’s Exact tests were used to test the bivariate associations between qualitative variables. The deviation from the Hardy–Weinberg equilibrium of the genotype distribution was performed using an exact Chi-Squared test from “SNPassoc” R package [54]. We also tested if the association between weight-status and variants of the CHIT1 gene remained significant after adjusting for age (defined as dichotomous variable based on median age) using the Generalized Cochran–Mantel–Haenszel test. We used the Student-*t* test or Mann–Whitney U test to identify significant differences in distributions of quantitative variables between two independent samples. Univariate analysis of variance (ANOVA) or Kruskal–Wallis test were used to compare multiple independent subgroups followed by post-hoc analysis for all pairwise comparisons using Tukey’s HSD or Dunn’s test when appropriate.

Multiple linear regression was applied to test the relation between BMI-for-age z score and log-transformed CHIT1 plasma activity. Clinical characteristics and the gene variants were the input data in multiple regression whenever they showed statistical significance (*p*-value < 0.05) or a tendency to statistical significance (0.05 < *p*-value < 0.10). Despite its statistical significance, the *G102S* genotype was included in the multiple regression if *dup24* proved statistically significant due to a cumulative effect reported in heterozygous for the *dup24* and *G102S* alleles depending on specific allelic loci of the two variants [34]. Univariate linear regression was used to test the link between potential predictors and log-transformed CHIT1 plasma activity. The unstandardized partial regression coefficient (β) was used to estimate the percentage change in CHIT1 plasma activity. A multivariable linear analysis was performed to predict change in CHIT1 plasma activity by independent predictors controlling the effect of demographic (age and gender) and clinical variables (time since weight gain) as possible covariates. The goodness-of-fit for multivariable regression model was assessed by adjusted R-squared, F-test of overall significance, residual standard error (RSE), and prediction error rate (defined as the ratio between RSE and the average value of the dependent variable).

All statistical tests used in data analysis were two-sided tests, a significant result being achieved if the *p*-value < 0.05.

## 3. Results

Seventy-one patients were eligible for our study. All patients with no detectable CHIT1 plasma activity were homozygous for *dup24* (3/71; 4.23%) and were excluded from the analysis.

### 3.1. Description of the Study Sample

The study sample consisted of 32 girls (47.1%) with a mean age of 12.47 ± 3.71 years and 36 boys (52.9%) with a mean age of 11.93 ± 3.18 years. The distribution of patients according to demographic, anthropometric, and clinical characteristics is presented in Table 2. Age distribution differed significantly across weight-status subgroups (Table 2), with the obese patients having a significantly higher mean age than the extremely obese (post-hoc Tukey′s HSD test, adjusted *p* = 0.0001), as shown in Table 2.

### 3.2. Association of Plasma-Uncorrected (CHIT1) and -Corrected (CHIT1-Corrected) Chitotriosidase Activity and Weight-Status Subgroups

**Figure 1:**
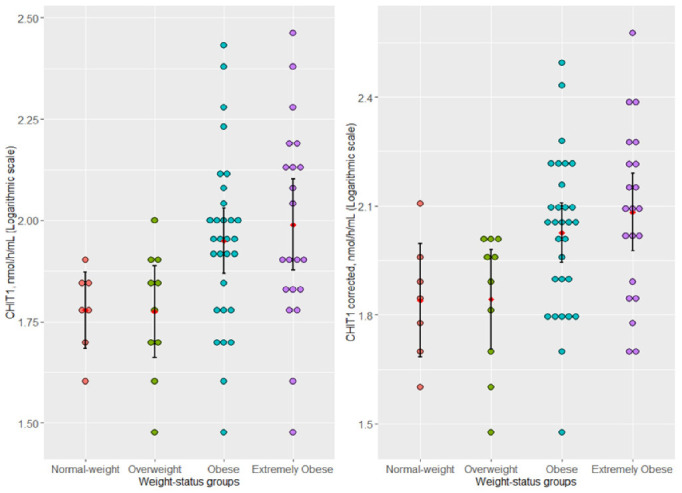
CHIT1 plasma activity across weight-status subgroups. Dot plots showing log-transformed CHIT1 activity (logCHIT1) and CHIT1-corrected plasma activity (logCHIT1-corrected) in the weight-status sugroups. Red points and black lines represented arithmetic means of log-transformed data with associated 95% confidence intervals (CI). Significant differences in logCHIT1 (ANOVA test: *p* = 0.0185) and logCHIT1-corrected (ANOVA test: *p* = 0.009) between overweight and extremely obese subgroups (Tukey’s HSD, adjusted; *p* = 0.048 for the logCHIT1 and *p* = 0.026 for the logCHIT1-corrected)..

The CHIT1 plasma activity (logCHIT1) and log-transformed CHIT1-corrected plasma activity (logCHIT1-corrected) showed higher values in children with obesity and extreme obesity (Figure 1). The post-hoc analysis showed a significant difference between overweight and extremely obese subgroups with higher values of logCHIT1 in patients with extreme obesity (geometric mean, 95% confidence interval (CI): 97.61, 95% CI: [75.3, 126.0] in extremely obese vs. 59.6, 95% CI: [46.0, 77.2] in overweight). No significant difference in logCHIT1 was found between the subgroup with extreme obesity and the control group (adjusted *p* = 0.110) or between children with extreme obesity and children with obesity (adjusted *p* = 0.908). We also noticed a marginal significance between logCHIT1-corrected in children with extreme obesity and the control group (adjusted *p* = 0.058), with higher mean values of logCHIT1-corrected in the extremely obese subgroup (geometric mean, 95% CI: 121.0, 95% CI: [94.6, 155.0] in extremely obese vs. 69.2, 95% CI: [48.5, 99.0] in the control group).

### 3.3. Genotype Distribution of dup24 and G102S Gene Polymorphisms in Weight-Status Subgroups

Homozygote (*wt/wt*), heterozygote (*wt/dup24)*, and mutant homozygote genotypes (*dup24/dup24*) have been identified in the evaluated children. The genotype distribution for both genetic variants followed the Hardy–Weinberg equilibrium in the control group (*p*-values > 0.05). No significant association between *G102S* polymorphism and weight-status subgroups within the studied genetic inheritance models (codominant, dominant, and allelic model) was identified. The frequency of the wild genotype (CC) was similar in each studied subgroup (Table 3). A similar result was found regarding the association between *dup24* and weight-status subgroups (*p*-values > 0.05).

### 3.4. Association of Plasma CHIT1 Activity with dup24 and G102S Polymorphisms within Weight-Status Subgroups

Considering *dup24*, a significant difference in the means of logCHIT1 plasma activity between wild type homozygote and heterozygote patients (*p* = 0.0003) was identified in the whole sample (n = 68), with the former subgroup having higher values (geometric mean, 95% CI: 93.0, 95% CI: [81.2, 107.0] vs. 55.2, 95% CI: [45.1, 67.5]). After stratifying by the weight-status, the difference remained significant for children with normal weight (Student *t*-test, *p* = 0.035), overweight (Student *t*-test, *p* = 0.006), extreme obesity (Student *t*-test, *p* = 0.007), with a marginal significance in obese patients (Student-*t* test, *p* = 0.056). Heterozygote patients had a lower value of logCHIT1 plasma activity compared to wild type homozygote patients in each weight-status subgroup: geometric mean, 95% CI: 49.3, 95% CI: [29.8, 81.7] vs. 619.6, 95% CI: [57.8, 84.0] in the normal-weight subgroup, 39.2 95% CI: [20.7, 74.0] vs. 73.4, 95% CI: [58.1, 87.7] in the overweight subgroup, 63.1, 95% CI: [39.2, 102.0] vs. 97.0, 95% CI: [79.4, 118] in the obese subgroup, and 66.5, 95% CI: [53.3, 82.9] vs. 104.1, 95% CI: [77.6, 140] in the extremely obese subgroup.

No significant differences in CHIT1 plasma levels were identified between wild type (CC) and variant (CT + TT) genotypes for *G102S* gene polymorphism, neither in the entire study group (Student *t*-test, *p* = 0.192), nor in each weight-status subgroup (Student *t*-test, *p*-values > 0.05).

### 3.5. The Effect of the dup24, G102S Polymorphism, and BMI-for-Age z Score upon Variation of CHIT1 Plasma Activity in Children with Overweight, Obesity, and Extreme Obesity

A significant positive linear association of logCHIT1 plasma activity with BMI-for-age z score and negative with *dup24* and *G102S* genotype (Table 4) was found in univariate regression analysis.

Given that a significant difference in logCHIT1 activity was found only between the initial overweight and extremely obese subgroups, the control group was not included in the multivariable model. In multivariable regression analysis, BMI-for-age z score, *dup24*, and *G102S* polymorphism remained significant independent predictors for CHIT1 plasma activity. A higher effect on the variation of logCHIT1 activity was explained by *dup24* (Table 4).

## 4. Discussion

Our results showed that CHIT1 plasma activity increases with the level of obesity in the evaluated children. More specifically, the chitotriosidase (CHIT1) circulating levels are significantly higher in children with extreme obesity than with overweight. The variation of logCHIT1 circulating levels is positively linearly explained by increas-ing the BMI-for-age z score with an amplified effect when covariates (*dup24*, *G102S*, age, gender, and time since weight gain) are controlled. In most of the evaluated children, the distribution of gender was homogenous, but extreme obesity was more frequent in prepubertal boys (Table 2). Our results are similar to previously reported findings on children from 11 to 14 years old in Cluj-Napoca [55] and Romania [56]. 

### 4.1. Association of Plasma Chitotriosidase Activity and Weight-Status Subgroups

We showed that children with normal weight and overweight have a lower circulating CHIT1 activity than children with obesity and extreme obesity (Figure 1), with the significance threshold being achieved when children with overweight were compared to those with extreme obesity. Furthermore, the corrected CHIT1 activity captures better the differences between the weight-status subgroups (Figure 1). This result suggests an extent of the inflammation with the grade of obesity based on previous findings that have indicated an association between CHIT1 activity and inflammatory conditions [14,15,57,58,59], probably mainly due to its biological role in reflecting macrophage activation [14]. The increasing CHIT1 activity has been attributed to macrophage activation in adults with obesity-driven complications and in children with glucose intolerance [23,25]. 

In addition to these results, our data indicate that CHIT1 may be a valuable measure for the extent of the adipose tissue inflammation even before obesity-driven complications occur in children. By contrast, inflammatory markers WBC levels, ESR, hCRP, and ferritin values did not increase with obesity levels in our patients (Table 2). These inflammatory markers, commonly evaluated in clinical practice, have been reported to have significantly elevated levels in children and adults with obesity and with complications such as metabolic syndrome and type 2 diabetes [60,61]. Consequently, our findings may also suggest that CHIT1 activity could be a more sensitive indicator for the initiation of inflammatory processes in children with obesity than the previous markers. The CHIT1 activity had been established as a sensitive biomarker and its reliability in reflecting the disease activity of various chronic inflammatory conditions has been proven [41,62,63]. In this context, our results open a promising new avenue of study focused on its role as an indicator of inflammatory status in children with BMI imbalance. Additionally, the attempts to facilitate the measurement of CHIT1 activity through blood spot tests [26,64,65,66,67] may be an incentive for evaluating it as a screening tool for complications in children with obesity.

### 4.2. Dup24 and G102S Gene Polymorphisms and CHIT1 Activity

The similar allelic and genotypic distribution of *dup24* and *G102S* polymorphism across the evaluated weight-status subgroups (Table 3) supports the appropriateness of the comparisons. Slightly similar genotype frequencies of the two CHIT1 gene variants compared with reported results in the European population were identified in our study. We found a smaller percentage of *dup24* heterozygous (21.13% in our study vs. 35% [30,32]) and homozygous (4.23% vs. and 6% to 10% [30,32]). Regarding *G102S,* 57.75% of our patients were heterozygotes and 12.68% were homozygotes compared to the reported frequencies of 41.7% and 3.5% [34]. These similarities might suggest that our findings may apply to children of European ancestry.

In accordance with previous studies accounting for the real effect of the 24 base pair duplication and *G102S* polymorphism upon CHIT1 activity in adults [27,28], heterozygosity for the *dup24* was associated with a significant decrease of its enzymatic activity in our patients when using 4-MU-3C as a substrate for the plasma activity measurement. *G102S* allele may also influence, although to a lesser extent than *dup24*, the measured CHIT1 activity in overweight, obese, and extremely obese pediatric patients (Table 4). As other findings have shown [28,32,59], our results indicate that identifying these two gene variants may be important when interpreting the CHIT1 enzymatic activity.

Regarding the effect of the evaluated gene variants, when the Bussink et al. [34] correction method was applied, CHIT1 enzymatic activity helped us differentiate normal weight from extremely obese patients in addition to the significant difference in the mean uncorrected CHIT1 activity between overweight and extremely obese children (Figure 1). Thus, a significant effect in the population is expected that should be validated in a larger sample. The cumulative effect that may appear when *dup24* is accompanied by *G102S* homozygous [34] might have influenced our results. Since no determination of the parental origin or alleles was done, the specific allelic loci of the two variants could not be evaluated. Therefore, the correction factor in patients heterozygous for the *dup24* and *G102S* alleles was not applied in our study. This limitation was partially overcome by including both gene variants as independent predictors in the multivariable model. Nevertheless, in addition to the *dup24* and *G102S*, other gene variants with previously reported negligible prevalence may affect the biochemical variability of the CHIT1 enzymatic activity [32].

### 4.3. Multivariable Analysis with CHIT1 Plasma Activity as the Dependent Variable

Our results suggest that BMI-for-age z score predicts the increase of CHIT1 circulating activity starting from children with overweight to children with extreme obesity after controlling *dup24* and *G102S* genotypes as covariates. Children with overweight, obesity, and extreme obesity who are also heterozygotes for *dup24* allele tended to have a lower value of *log* CHIT1 plasma activity (a decrease by 39.74% in the CHIT1 plasma activity, Table 4). Children carrying the CT+TT genotype had a decrease by 25.87% of CHIT1 plasma activity compared with patients carrying the CC genotype (Table 4). The presence of *dup24* allele led to a decrease by 36.90% of CHIT1 plasma activity when age, gender, and time since weight gain were evaluated as covariates. 

Gender-specific body fat disposition closely influences the degree of inflammation in obese adults, with a predominance of visceral fat in males, eliciting a greater inflammatory response than in females [68,69]. Similarly, a more accelerated increase in central fat was reported in boys than in girls [70]. Aging is also known to affect in adults the levels of pro-inflammatory markers, such as interleukin 6 (IL-6) or tumor necrosis factor-α (TNF-α) [71]. Contrary to the proven effect of age and gender upon inflammation in the adult population, no significant effect of age or gender upon the CHIT1 circulating activity was found in our study (Table 4). Age did not predict any change in CHIT1 activity in our patients in the multivariable model. Still, our results should be interpreted cautiously and confirmed in a larger sample, as children with extreme obesity were younger than those with overweight. Although other potential confounding variables may influence the CHIT1 activity, such as smoking (active or passive) or comorbidities, previous findings showed a less important effect over the pro-inflammatory status in children than in adults [60,72,73,74].

### 4.4. Study Limitations and Further Research

The relatively small sample size and the study′s cross-sectional nature conducted in an exploratory manner limit the generalizability of our results. The difference in CHIT1 activity between the control and weight-status subgroups might prove statistically significant in larger samples. Apart from the small sample size of the control group, the inclusion of the normal-weight children that were addressed to the hospital with possible subclinical inflammation might have influenced our findings. Sample size estimation would have limited our study′s risk of type II error. However, post-hoc power analysis of our linear multiple regression model achieved a power > 0.99 (effect size f^2^ = 0.52), so our results are unlikely to be underpowered.

Considering that the percentage of parents who incorrectly estimate their children′s weight varies from 11.9% for children with normal weight to 41.5% for children with overweight or obesity, the evaluation of time since weight gain by the parents might be subjective to reporting errors [55]. A longitudinal follow-up evaluation of disease duration would allow a more appropriate assessment of obesity progression [75]. The influence of the waist-to-hip ratio (WHR) over CHIT1 plasma activity could not be evaluated due to different protocols applied in the healthcare units where our study was conducted. However, waist-to-hip ratio had a higher explanatory ability for increasing inflammatory markers than BMI in adults [76]. In addition, WHR has been previously reported to be associated with inflammation markers, such as high-sensitivity C-reactive protein, TNF-α, amyloid A, and interleukine-6, in children affected by obesity-driven inflammation [77]. As a consequence, WHR or the aforementioned indicators might be more appropriate than BMI-for-age z score for grouping children according to the level of obesity. Further research should also evaluate the association of CHIT1 activity with measures of central adiposity and the aforementioned biomarkers. The accuracy of circulating CHIT1 activity levels as an indicator of CHIT1 local expression in adipose tissue macrophages is still debated [15] and should be validated in further studies.

## 5. Conclusions

Our results showed that CHIT1 plasma activity increases with the obesity level in our pediatric population without metabolic complications. More specifically, the elevated CHIT1 circulating levels are significantly higher in children with extreme obesity than in children with overweight. The variation in logCHIT1 circulating levels is positively linearly explained by increasing the BMI-for-age z score with an amplified effect when covariates (*dup24*, *G102S*, age, gender, and time since weight gain) are controlled. Validation of the present findings by future investigations with a larger cohort is necessary to acknowledge the effect of the 24-base pair duplication and *G102S* polymorphism of CHIT1 gene when evaluating CHIT1 activity as an inflammatory marker in children with obesity.

## Figures and Tables

**Table 1:** Primer sequences for identification of *dup24* in the CHIT1 gene.

Forward Primer 5′→3′ Sequence	Reverse Primer 5′→3′ Sequence
GAAGAGGTAGCCAGGCTCTGG	CTGCCGTAGCGTCTGGATGAG

**Table 2:** Distribution of clinical and laboratory characteristics in weight-status subgroups.

Variable	Weight-Status Subgroups				
	Normal Weight (n1 = 7)	Overweight (n2 = 10)	Obesity (n3 = 30)	Extreme Obesity (n4 = 21)	*p*-Value
Clinical characteristics					
Gender					0.061
Girls	3 (42.86)	8 (80.00)	15 (50.00)	6 (28.57)	
Boys	4 (57.14)	2 (20.00)	15 (50.00)	15 (71.43)	
Tanner staging ^(a)^					0.023 ^#^
prepubertal	1 (20.00)	3 (33.33)	4 (15.38)	10 (55.56)	
pubertal	2 (40.00)	1 (11.11)	19 (73.08)	8 (44.44)	
postpubertal	2 (40.00)	5 (55.56)	3 (11.54)	0 (0.00)	
Age (years)	12.86 ± 4.38	12.63 ± 2.93	13.64 ± 2.69	9.66 ± 3.00	0.0002
Time since weight gain ^(b)^ (years)	na	8 (3.78–11.66) {2.17–17.64}	5.18 (3.44–7.85) {0.25–17.52}	4.9 (3.59–6.53) {2.09–11.98}	0.008
Inflammation-related parameters					
hCRP ^(c)^ (mg/dL)	0.28 (0.25–0.29) {0.23–0.29}	0.24 (0.21–0.24) {0.20–0.31}	0.27 (0.26–0.30){0.18–1.23}	0.23 (0.21–0.29) {0.14–0.31}	0.600
Ferritin ^(d)^ (ng/mL)	34.20 (29.9–38.5) {25.6–42.8}	36.95 (28.0–44.3) {21.3–49.2}	40.9 (19.83–6.48) {12.9–92.8}	27.2 (25.1–41.4) {20.4–47.5}	0.981
WBC ^(e)^ (×103/μL)	5.54 (4.66–5.77) {4.41–7.18}	8.11 (7.21–8.65) {6.53–10}	7.27 (6.52–7.91) {6.11–8.24}	7.82 (7.2–9.18) {6–9.84}	0.002
ESR ^(f)^ (mm/h)	7 (7–8.5) {3–16}	16.6(12–22) {6–30}	13.2 (6–13) {4–36}	12 (9–15) {3–19}	0.432

Data were presented as absolute (relative) frequencies or arithmetic, mean ± standard deviation or median (Q1–Q3) {Min–Max}; ^(a)^ complete case data n = 57; ^(b)^ complete case data n = 61; ^(c)^ complete case data n = 30; ^(d)^ complete case data n = 18; ^(e)^ complete case data n = 34; ^(f)^ complete case data n = 27; asymptotic or exact *p*-values obtained from Chi-square test, Fisher’s exact test or Kruskal–Wallis test; ^#^ simulated *p*-value of Fisher’s exact test based on 2000 replicates; na: not applicable. WBC = total absolute white blood cells count; hCRP = human C-reactive protein; ESR = erythrocyte sedimentation rate.

**Table 3:** Allelic and genotypic distribution of *dup24* and *G102S* polymorphism across weight-status subgroups.

Gene Variant	Normal Weight (n1 = 7)	Overweight (n2 = 10)	Obesity (n3 = 30)	Extreme Obesity (n4 = 21)	*p*-Value	Adjusted *p*-Value
*G102S* (rs2297950)						
CC ^[C]^	4 (57.14)	2 (20.0)	10 (33.33)	5 (23.81)	0.428	0.728
CT	2 (28.57)	8 (80.0)	16 (53.33)	12 (57.14)		
TT	1 (14.29)	0 (0.0)	4 (13.33)	4 (19.05)		
CT + TT ^[D]^	3 (42.86)	8 (80.0)	20 (66.67)	16 (76.19)	0.370	0.161
C ^[A]^	10 (71.43)	12 (60.0)	36 (60.0)	22 (52.38)	0.641	0.319
T	4 (28.57)	8 (40.0)	24 (40.0)	20 (47.62)		
HWE (*p*-value)	0.441	0.173	0.711	0.675		
*dup24* * (rs3831317)						
*wt/wt* ^[a]^	4 (57.14)	7 (70.0)	24 (80.0)	18 (85.71)	0.380	0.965
*wt/dup24* ^[b]^	3 (42.86)	3 (30.0)	6 (20.0)	3 (14.29)		
*wt* allele	11 (78.57)	17 (85.0)	54 (90.0)	39 (92.86)	0.407	0.967
*dup24* allele	3 (21.43)	3 (15.0)	6 (10.0)	3 (7.14)		
HWE (*p*-value)	0.471	0.577	0.543	0.012		

Data were presented as absolute (relative) frequencies; ^[C]^ Codominant genetic models (CC = wild genotype vs. CT = heterozygote genotype vs. TT = mutant genotype); ^[D]^ Dominant model (CT + TT vs CC genotype); ^[A]^ Allelic model (C allele vs. T allele); HWE: Hardy–Weinberg Equilibrium; *p*-values were obtained from Fisher’s Exact test or Chi-squared test; adjusted *p*-values were obtained from Generalized Cochran–Mantel–Haenszel test; * homozygotes for *dup24* were not included in the analysis; ^[a]^ *wt*: wild type allele; ^[b]^ *dup24*: 24 base pair duplication allele.

**Table 4:** Univariable and multivariable linear regression model for significant predictors of log-transformed chitotriosidase plasma activity (n = 61).

**Variables**	**Unadjusted b** **[95% CI]**	***p***	**Adjusted b** **[95% CI]**	**Adjusted β** **[95% CI]**	***p***
logCHIT1 plasma activity					
BMI z score	0.08 [0.03; 0.13]	0.003 *	0.06 [0.01; 0.12]	0.30 [0.03; 0.57]	0.031 *
*dup24* (*wt/dup24* vs. *wt/wt*)	−0.22 [−0.36; −0.09]	0.002 *	−0.25 [−0.38; −0.12]	−1.10 [−1.66, −0.55]	<0.001 *
*G102S* (CT + TT vs. CC)	−0.13 [−0.26; −0.003]	0.045 *	−0.19 [−0.30; −0.08]	−0.83 [−1.32, −0.35]	0.001 *
Age (months)	−0.0008 [−0.002; 0.0007]	0.281	0.0002 [−0.001; 0.002]	0.04 [−0.23; 0.30]	0.787
Gender (male vs. female)	0.08 [−0.03; 0.20]	0.159	0.05 [−0.06, 0.15]	0.21 [−0.24; 0.65]	0.361
Time since weight gain (months)	−0.01 [−0.03; 0.004]	0.142	−0.003 [−0.02, 0.01]	−0.05 [−0.29; 0.18]	0.649

CC = wild genotype vs. CT = heterozygote genotype vs. TT = mutant genotype; Multivariable Model performance: F-test of overall model significance: F(6,54) = 6.18, *p* < 0.00005. Adjusted multiple correlation coefficient: R^2^ = 0.341, RSE = 0.185, prediction error = 9.54%. b = unstandardized partial regression coefficients; β = standardized partial regression coefficient; 95% CI = 95% Confidence level; RSE = Residual standard error; * statistical significance: *p* < 0.05.

## Data Availability

The raw data analyzed in this study are part of a PhD study and can be obtained upon reasonable request addressed to Ioana Țaranu (taranu.ioana@umfcluj.ro).
